# Single-cell and transcriptomic analyses reveal the role of PCDH17 in the non-inflammatory tumor microenvironment of pancreatic cancer

**DOI:** 10.3389/fendo.2025.1559909

**Published:** 2025-05-23

**Authors:** Yong Sun, Hong Wan, Jie Xiong, Kun Cao, Dashuai Yang, Jun Huang

**Affiliations:** ^1^ Department of Hepatobiliary and Pancreatic Surgery, Hefei First People’s Hospital, Third Affiliated Hospital of Anhui Medical University, Hefei, Anhui, China; ^2^ Department of General Surgery, The First Affiliated Hospital of Anhui Medical University, Hefei, Anhui, China; ^3^ Department of Hepatobiliary Surgery, Renmin Hospital of Wuhan University, Wuhan, China

**Keywords:** PCDH17, pancreatic cancer, immunotherapy, non-inflammatory, tumor microenvironment

## Abstract

**Background:**

The PCDH17 family has emerged as a prominent research focus in the field of oncology; however, the precise mechanism underlying the regulatory role of PCDH17 in shaping the inflammatory tumor microenvironment in pancreatic cancer remains elusive.

**Method:**

A thorough examination was carried out to explore the presence of PCDH17 in cancerous tumor tissues and its association with genes related to inflammation. Additionally, we comprehensively examined the association between PCDH17 and immune cell infiltration as well as immunotherapy in pancreatic cancer. Moreover, the single-cell data of pancreatic cancer was utilized for analyzing PCDH17 expression, cell differentiation, and intercellular communication.

**Result:**

PCDH17 exhibited differential expression across various tumor types. The low-expression group of PCDH17 showed reduced levels of inflammatory genes. Immunoinfiltration analysis indicated a significant association between T-cell infiltration and the expression of PCDH17. Analysis of single-cell sequencing data revealed that PCDH17 was primarily expressed in endothelial cells, with a decrease in expression observed during cellular differentiation trajectory. Notably, inflammation-related genes also displayed significant changes in their expression patterns along the endothelial cell differentiation trajectory. Cellular communication investigations unveiled multiple signaling pathways through which endothelial cells interacted with T cells. The presence of PCDH17 in endothelial cells was verified through various immunofluorescence techniques, and a simultaneous decline in its levels was observed alongside the decrease in inflammatory factors within the tumor microenvironment.

**Conclusion:**

The predominant expression of PCDH17 was observed in endothelial cells, exhibiting a strong association with inflammatory genes and infiltration of immune cells. PCDH17 exhibits potential as a target for regulating the immune-suppressive tumor microenvironment in pancreatic cancer.

## Background

Pancreatic cancer patients exhibit an exceedingly unfavorable prognosis, characterized by a five-year survival rate of less than 10% (1, 2). The biology of pancreatic cancer exhibits distinct characteristics in comparison to other malignant tumors (3). Firstly, the absence of distinct clinical symptoms in patients with pancreatic cancer contributes to a significantly low rate of early diagnosis (4, 5). Secondly, pancreatic cancer presents a highly pronounced pathological feature of distant metastasis, with the majority of patients exhibiting either intra-pancreatic or remote organ metastases upon initial diagnosis (6). Finally, pancreatic cancer is characterized by a profoundly debilitating constitution, with the majority of patients experiencing concurrent cachexia (7). The aforementioned statement significantly diminishes the likelihood of patients undergoing surgical intervention and enduring chemotherapeutic agents (8).

The tumor microenvironment of pancreatic cancer exhibits a distinctive and intricate nature (9, 10). It encompasses not only stromal cells and immune cells but also disseminates a plethora of inflammatory mediators (11). It is well-established that immune cells play a pivotal role in executing immune functions. However, recent studies have shed light on the equally significant involvement of stromal cells, particularly endothelial cells, in regulating immune function (12). The characteristics of chronic inflammation and pancreatic cancer exhibit remarkable similarities, characterized by a pronounced proliferation of fibrous tissue within the interstitium (13). Concurrently, the expression levels of invasive factors and growth factors are heightened in both inflammatory conditions and pancreatic malignancies (14, 15). The role of inflammatory factors IL-1b and IL-8 in tumor cell proliferation and metastasis has been demonstrated through their regulation of apoptosis within the tumor microenvironment (16, 17).

Procalcitonin, a transmembrane protein belonging to the calreticulin superfamily, plays a crucial role in cell adhesion and the regulation of downstream signaling pathways (18). The involvement of procalcitonin in the occurrence and progression of tumors has been demonstrated by research findings (19, 20). However, the underlying mechanism of PCDH17 in pancreatic cancer remains elusive. This study aims to elucidate the precise mechanism by which PCDH17 modulates the non-inflammatory tumor microenvironment in pancreatic cancer.

## Method

### Data download and collation

Tumor mutation data and tumor RNA sequencing data (FPKM values) were acquired from the UCSC Xena data site. The RNA-seq data underwent a logarithmic transformation, while somatic mutation data were processed using VarScan2 to determine TMB. Furthermore, the research integrated single cell sequencing findings from a dataset called GSE212966, which encompassed 6 instances of pancreatic cancer and 6 instances of adjacent non-cancerous tissue. **Supplementary Table 1** summarized the set of inflammatory genes.

### Pan-cancer analysis

In this study, transcriptome sequencing data from 33 tumors were compiled and analyzed to compare the expression of PCDH17 between tumor and normal groups. Additionally, the correlation between PCDH17 expression in these tumors and inflammatory factors was investigated. The single-sample gene set enrichment analysis (ssGSEA) algorithm was employed to evaluate immune cell infiltration patterns within the 33 tumors. In addition, the study investigated the correlation between PCDH17 expression and the level of immune cell infiltration. Lastly, an investigation was carried out to explore the connection between PCDH17 expression and immunotherapy.

### Characterization of inflammatory factors in the tumor microenvironment of pancreatic cancer

A compilation of 122 inflammatory factors from prior research studies was utilized. The expression levels of PCDH17 in pancreatic cancer were divided into two groups based on high and low expression. Analysis of correlation revealed variations in the expression patterns of the 122 inflammatory factors between the groups with high and low PCDH17 expression. The tumor immune cycle, a 7-step process involving the body’s immune function to eliminate tumor cells, was further investigated for potential effects of PCDH17 on its progression. Immune cell infiltration in pancreatic cancer was assessed using seven commonly applied algorithms: Cibersort-ABS, MCP-counter, quanTIseq, TIMER, xCell, TIP, and TISIDB. Previous research was used to compile a list of important genes in immune cells, which were then analyzed for differences in expression levels between groups with high and low PCDH17 expression. To evaluate the presence of T cell infiltration in pancreatic cancer, the Ayers algorithm was utilized and subsequently investigate the correlation between PCDH17 expression and T cell infiltration (21) **Supplementary Table 2**.

### The potential therapeutic role of PCDH17 in managing pancreatic cancer

The therapeutic potential of PCDH17 in pancreatic cancer was further explored by analyzing the IMvigor210 dataset, which provides patient information on immunotherapy. Furthermore, GSVA enrichment analysis was utilized to investigate the differences in cancer pathway activity between groups with high and low expression levels of PCDH17. Furthermore, we compiled a comprehensive list of commonly used immune checkpoint inhibitory genes based on previous studies and demonstrated their relationship with PCDH17. TISIDB database summarized the gene-drug interaction network, allowing us to predict potential drug targets for pancreatic cancer.

### Single-cell sequencing data processing

The dataset GSE212966 consisted of single-cell sequencing data obtained from 6 individuals with pancreatic cancer and 3 individuals with paraneoplastic tissue. The R programming language and appropriate function packages were employed to import the data and create Seurat objects. Quality control measures for the single-cell data included: (1) identification of cells with mitochondrial gene expression exceeding 5%, (2) exclusion of doublets, (3) removal of cells exhibiting erythrocyte gene expression over 3%, and (4) correction for cell cycle effects on downstream analysis. Subsequently, the ‘harmony’ package was employed to integrate the datasets for further investigation. The Unified Manifold Approximation and Projection (UMAP), a dimensionality reduction technique, visualized cell clustering in a two-dimensional graph, facilitating annotation of distinct cell subpopulations based on existing literature reports (12, 22–25).

### Analysis of inflammatory pathway activity

The genes associated with inflammation-related pathways were obtained from the GSEA website and evaluated for their inflammatory pathway scores in individual cells using the “AUCell” package. Subsequently, a comparison was made between the cellular inflammatory pathway scores of normal and tumor groups to identify any differences.

### Trajectory analysis

The Monocle2 algorithm utilizes unsupervised learning on the matrix of single-cell transcriptome expression to allocate cells into distinct branches of the developmental trajectory. Mimetic analysis allows for the inference of cell differentiation paths during the development or evolution of cell subtypes. Furthermore, we explored the relationship between PCDH17 expression and the developmental trajectory of cells. To visualize and group heatmaps based on inflammatory gene expression in different branches of the trajectory, a pseudotime heatmap plot was generated.

### Analysis of cellular communication

The cell transmits a message through the medium to another cell and interacts with the corresponding receptor of the target cell. This process, known as cellular signaling, induces a cascade of physiological and biochemical changes within the cell, ultimately resulting in biological effects on the entire cell. CellPhoneDB is an openly accessible database containing information on tissue receptors, ligands, and their interactions. CellChat employs cellular gene expression levels and characterizes the interactions between ligands and receptors to deduce intercellular communication pathways and measure their magnitude.

### Multiple immunofluorescence

Procure tumor and neighboring tissue specimens from patients undergoing curative pancreatic cancer surgery, followed by performing numerous immunofluorescence examinations to ascertain the exact localization of PCDH17 expression. Subsequently, investigate the potential relationship between CXCL12 and levels of PCDH17 expression.

### Statistical analysis

The associations between variables were assessed using Pearson or Spearman coefficients. For continuous variables that adhered to a normal distribution, comparisons were conducted using t-tests. Alternatively, the Mann-Whitney U test was employed for non-normally distributed continuous variables. Categorical variables were compared utilizing either the chi-square test or Fisher’s exact test. The statistical analysis was performed using R software (version 4.1.2, https://www.r-project.org/). Statistical significance was defined as P < 0.05 (two-tailed), with *denoting P < 0.05, **denoting P < 0.01, ***denoting P < 0.001, and ****denoting P < 0.0001.

## Result

### Pan-cancer analysis

**Figure 1:**
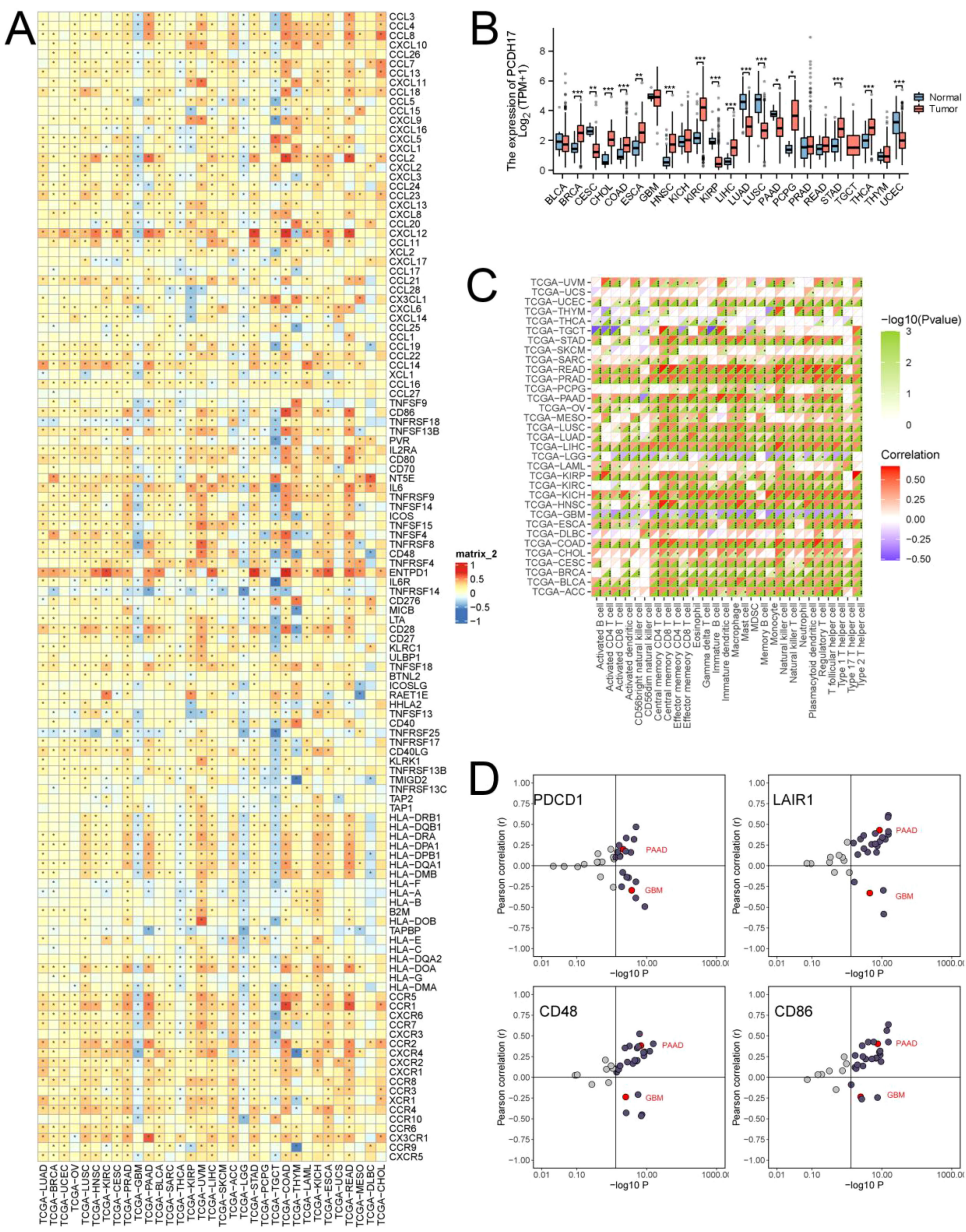
Comprehensive analysis of PCDH17 in pan-cancer. **(A)** Heat map of the relationship between PCDH17 and inflammation factor. **(B)** Differential analysis of PCDH17 expression. **(C)** Correlation analysis of PCDH17 immune cell infiltration. **(D)** Analysis of PCDH17 and immune checkpoint inhibitor genes correlations. * denoting P < 0.05, ** denoting P < 0.01, and *** denoting P < 0.001.

PCDH17 exhibited distinct patterns of expression across different tumor types, with upregulated expression observed in breast, cholangiocarcinoma, colon, and esophageal cancers. Conversely, decreased expression of PCDH17 was noted in cervical cancer, renal clear cell carcinoma, squamous cell lung cancer, and pancreatic cancer. The expression of PCDH17 showed inconsistent trends across different tumors, despite a strong correlation with 122 inflammatory factors. The analysis of immune infiltration revealed a strong association between PCDH17 and immune cells. Subsequently, the relationship between PCDH17 and immunotherapeutic target genes exhibited variability across different tumor types. These observations suggested the involvement of PCDH17 in the regulation of the tumor microenvironment associated with inflammation (**Figure 1**).

### Relationship between PCDH17 and inflammatory factors in pancreatic cancer

**Figure 2:**
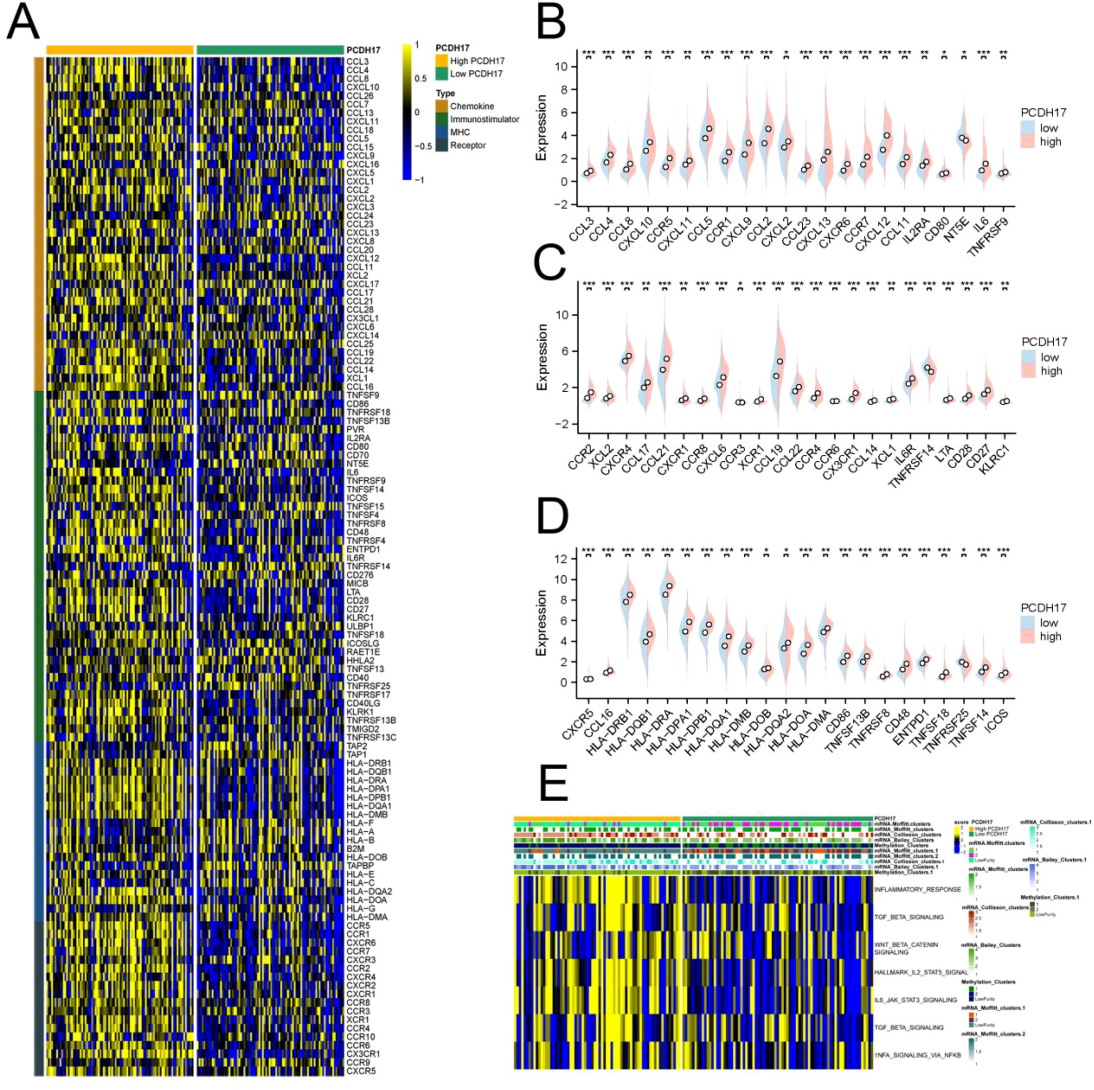
Comprehensive analysis of PCDH17 and inflammation factor in pancreatic cancer. **(A)** Heatmap of inflammation factor correlations in the PCDH17 high and low expression groups. **(B–D)** Differential analysis of inflammation factor in the PCDH17 high and low expression groups. **(E)** Enrichment analysis of inflammatory pathways in PCDH17 high and low expression groups. * denoting P < 0.05, ** denoting P < 0.01, and *** denoting P < 0.001.

The expression of inflammatory factors was found to be significantly elevated in the PCDH17 high-expression group compared with the PCDH17 low-expression group, suggesting a role for PCDH17 as an anti-inflammatory regulator within the tumor microenvironment of pancreatic cancer. The pathway enrichment analysis revealed that the PCDH17 high-expression group exhibited increased scores in multiple signaling pathways associated with inflammatory response, including TGF BETA signaling, IL2 STAT5 signaling, IL6 JAK STAT3 signaling, and TNFA signaling (**Figure 2**).

### PCDH17 strongly associated with the tumor microenvironment in pancreatic cancer

**Figure 3:**
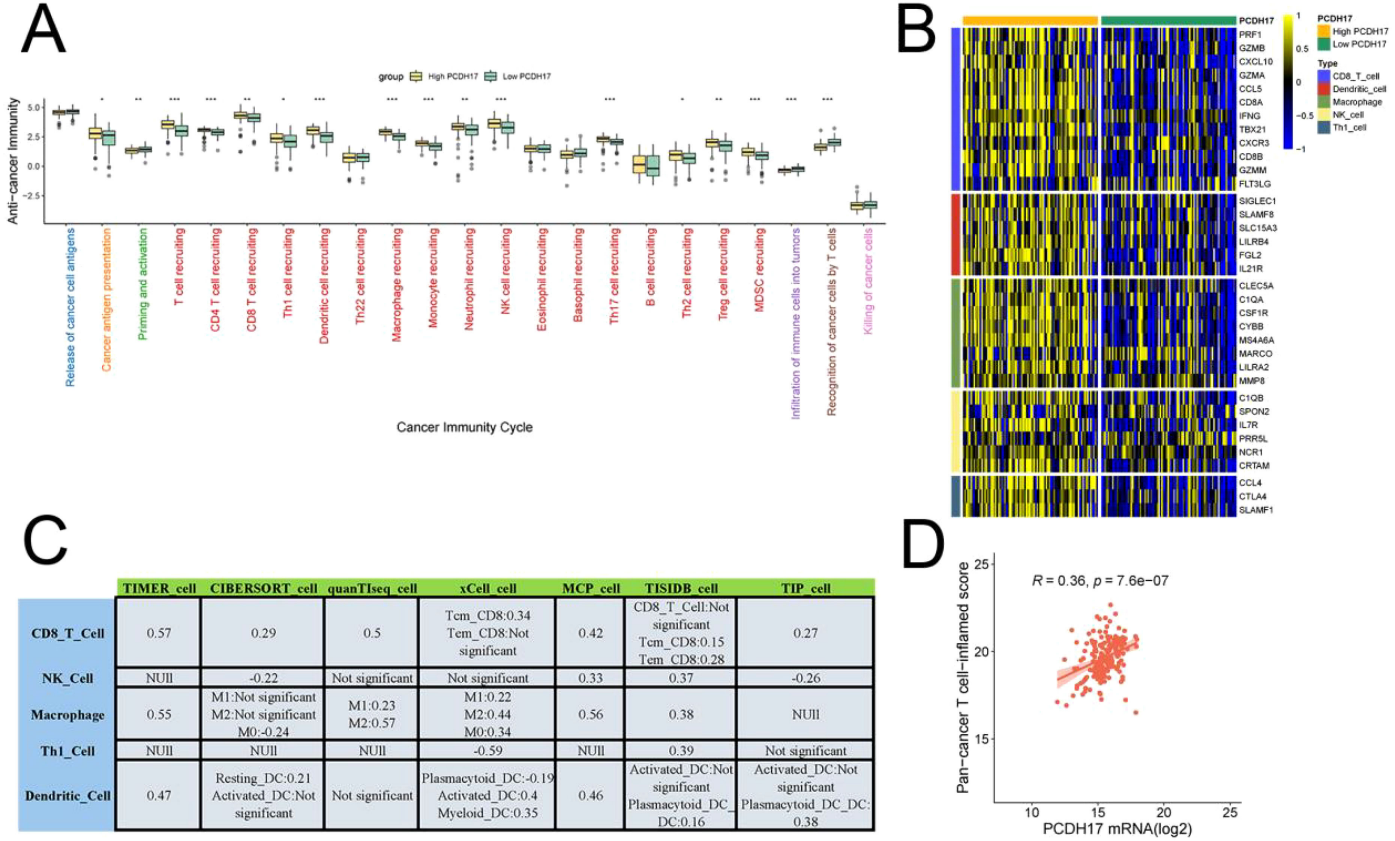
Comprehensive analysis of PCDH17 in the tumor microenvironment of pancreatic cancer. **(A)** Relationship between PCDH17 and the cancer immune cycle. **(B)** Differential analysis of gene expression for immune cell function in the high and low PCDH17 groups. **(C)** Seven algorithms to assess the relationship of PCDH17 to immune cell infiltration. **(D)** Analysis of PCDH17 and T-immune cell infiltration correlation. * denoting P < 0.05, ** denoting P < 0.01, and *** denoting P < 0.001.

PCDH17 was found to play a critical role in regulating immune events essential for tumor growth control. The high-expression group of PCDH17 demonstrated significantly elevated scores in immune-related processes, including cancer antigen presentation, T cell recruitment, CD8-positive T cell recruitment, and Treg cell recruitment, compared to the low-expression group. Immunoinfiltration analysis revealed a strong positive correlation between PCDH17 expression and the abundance of CD8-positive T cell infiltration. Additionally, results obtained using Ayers’ algorithm further confirmed that PCDH17 expression levels were positively correlated with T-cell infiltration abundance. Moreover, the high-expression group of PCDH17 exhibited increased expression levels of major functional genes in immune cells (**Figure 3** **).**

### Drug therapy

**Figure 4:**
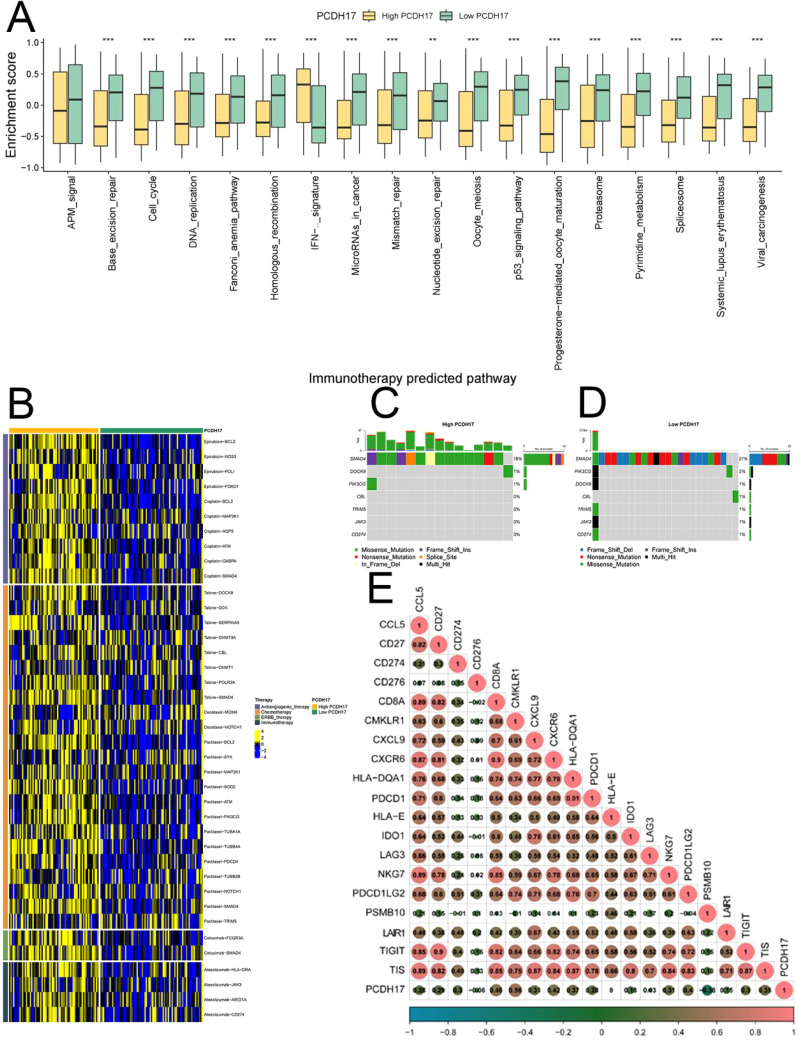
Analysis of PCDH17 in immunotherapy of pancreatic cancer. **(A)** Differential analysis of tumor-associated pathway enrichment in high and low PCDH17 expression groups. **(B)** Potential therapeutic drugs in the PCDH17 high and low expression group. **(C, D)** Tumor mutation burden analysis of the PCDH17 high and low expression group. **(E)** Correlation analysis of PCDH17 and immune checkpoint inhibitor genes expression. ** denoting P < 0.01, and *** denoting P < 0.001.

The high and low PCDH17 groups displayed significant variations in the scores of multiple tumor-related pathways, suggesting that PCDH17 plays a role in the progression of pancreatic cancer tumors. Utilizing drug-gene interactions sourced from the TISIDB database, this study made predictions regarding potential therapeutic agents for both the high and low PCDH17 groups. Expression differences were observed in the PCDH high and low expression groups for genes associated with multiple drug therapeutic targets. Tumor mutational loads of potential therapeutic drug target genes exhibited variations in both PCDH17 groups. Notably, a strong correlation was found between PCDH17 and immunotherapy inhibitor genes (**Figure 4**).

### Single-cell data processing

**Figure 5:**
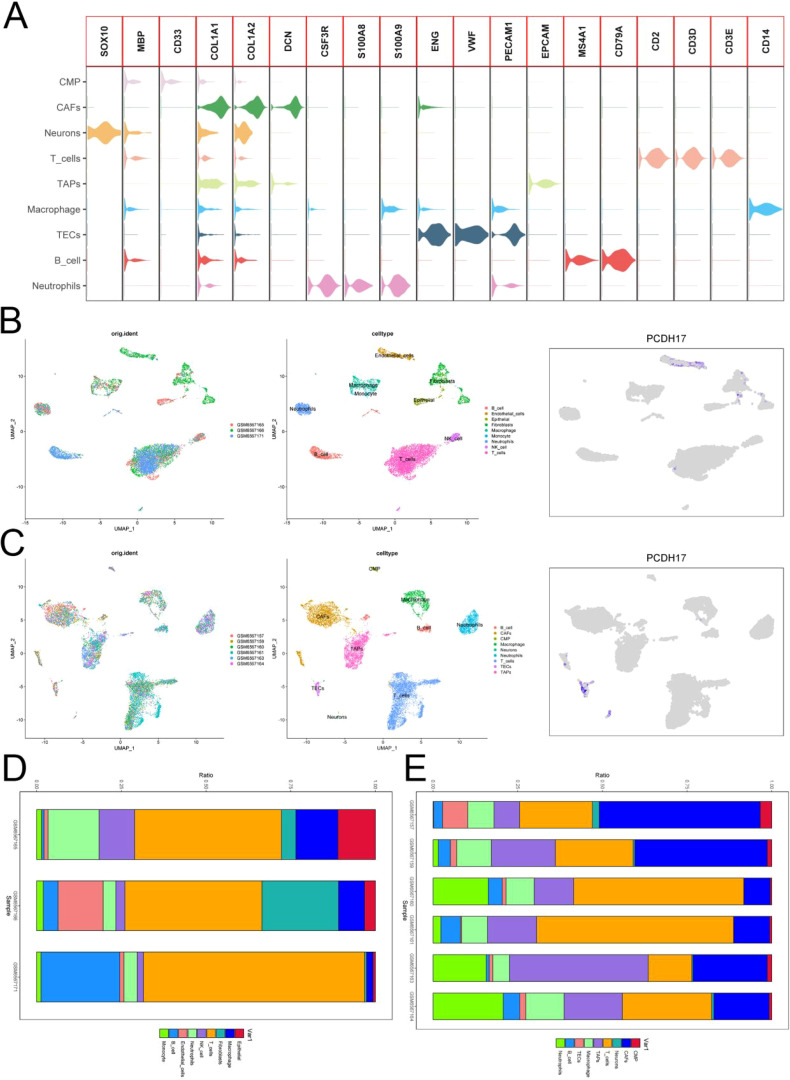
Single-cell analysis. **(A)** Annotation of cellular subpopulations. **(B)** UMAP plot presenting the results of single-cell data from three normal pancreatic tissues. **(C)** UMAP plot showing results of single-cell data from six pancreatic cancer tissues. **(D)** Proportion of cells in three normal pancreatic tissues. **(E)** Proportion of cells in six pancreatic cancer tissues. (TECs, tumor endothelial cells; TAPs, Tumor associated epithelial cells; CAFs, Tumor-associated fibroblasts).

The GSE212966 dataset yielded 11,864 cells from pancreatic cancer samples and 10,603 cells from pancreatic cancer paracellular tissues after excluding low-quality cells. We performed computational doublet removal using Scrublet during preprocessing (doublet score threshold = 0.25), which estimated an overall doublet rate of 8-12% - significantly lower than the typical tumor range due to our optimized cell loading concentration. Cell clusters were accurately classified into distinct cell types based on rigorous referencing of high-quality literature. Meanwhile, the UMAP plots were utilized to visually present the outcomes of data analysis. PCDH17 exhibited predominant expression in endothelial cells within the tumor microenvironment of pancreatic cancer, while its expression was nearly negligible in immune cells (**Figure 5**).

### Inflammation-related pathway analysis

**Figure 6:**
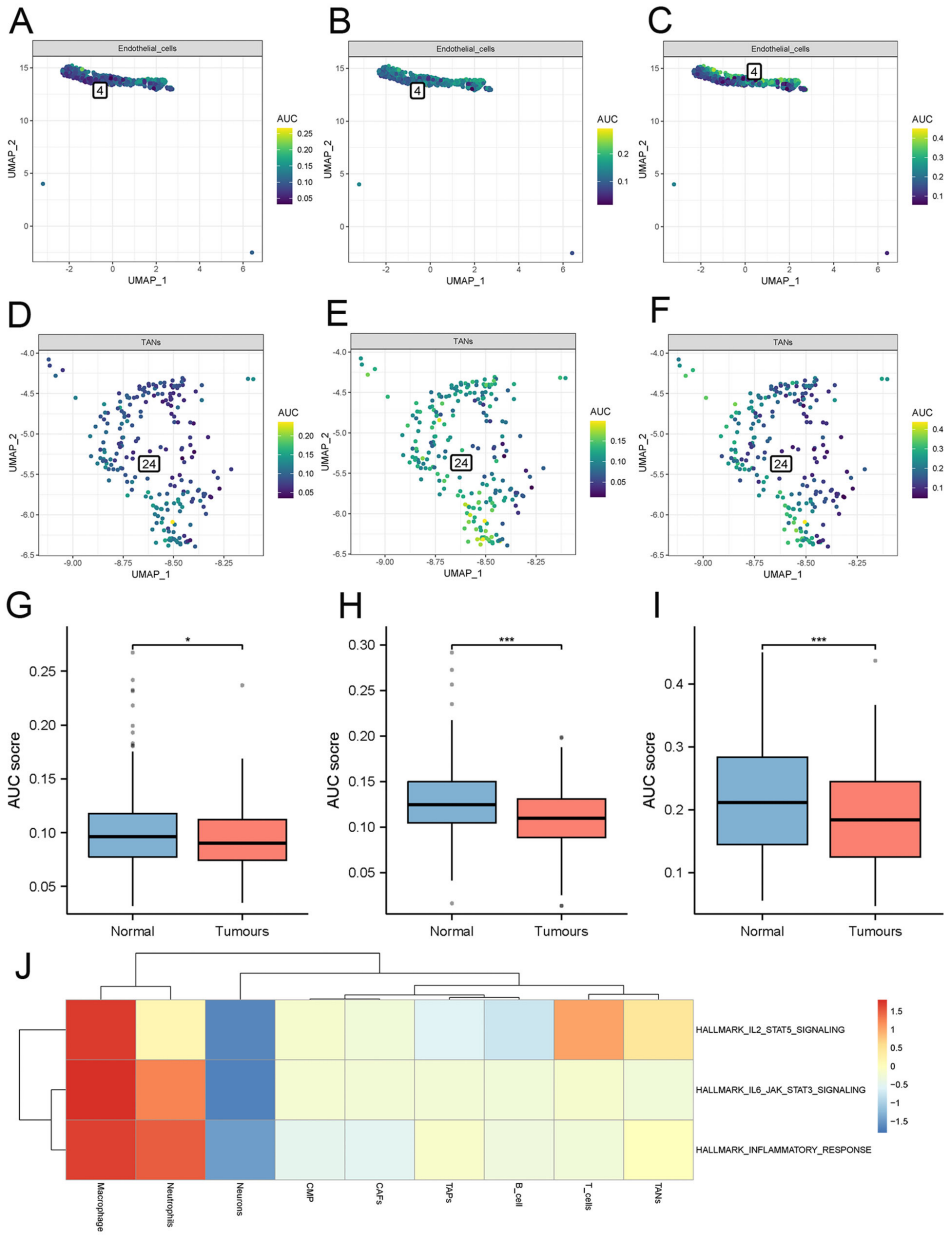
Analysis of inflammatory pathway activity. **(A–C)** Inflammatory pathways scores in normal endothelial cells. **(D–F)** inflammatory pathways scores in tumor-associated endothelial cells. **(G–I)** Differential analysis of three inflammatory pathway scores -inflammatory response signaling pathway, IL6 JAK STAT3 signaling pathway, and TNFA signaling pathway. **(J)** Single-cell GSVA enrichment analysis. (TECs, tumor endothelial cells; TAPs, Tumor associated epithelial cells; CAFs, Tumor-associated fibroblasts). * denoting P < 0.05, and *** denoting P < 0.001.

The comparison of results obtained from the “AUCell” package analysis on cellular immunity-related pathways revealed that the activities of the inflammatory response signaling pathway, IL6 JAK STAT3 signaling pathway, and TNFA signaling pathway were comparatively lower in the tumor group as compared to the normal group (**Figure 6**).

### Cell differentiation trajectory

**Figure 7:**
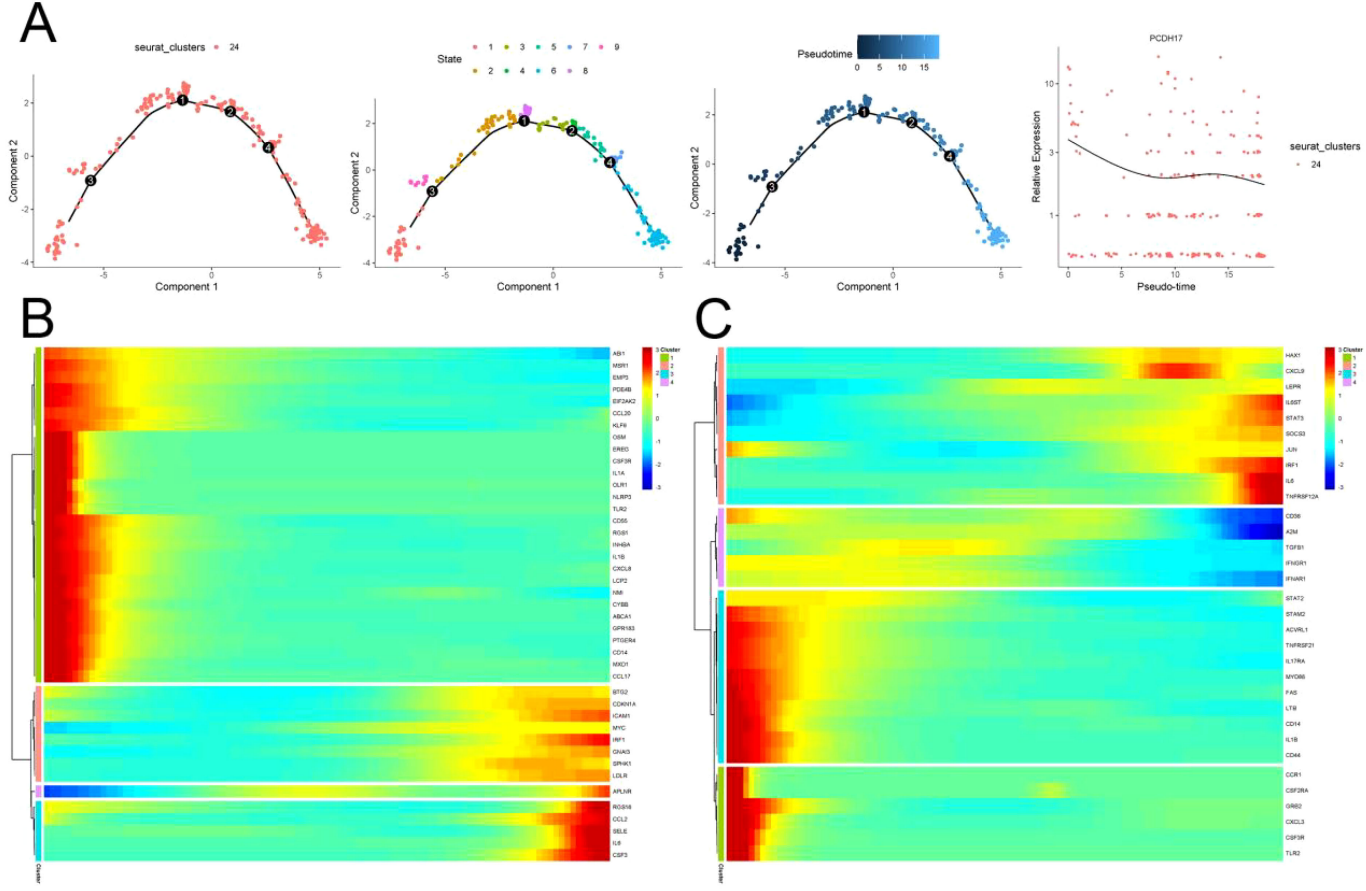
Trajectory analysis of endothelial cell differentiation in pancreatic cancer. **(A)** Trajectory of endothelial cell differentiation and changes of PCDH17 expression in pancreatic cancer. **(B, C)** Trends in the expression of the inflammatory pathways on inflammatory pathways differentiation trajectories. (TECs, tumor endothelial cells; TAPs, Tumor associated epithelial cells; CAFs, Tumor-associated fibroblasts).

Pseudo time series analysis was conducted on endothelial cells using the “Monocle” function of the R package. The differentiation of endothelial cells resulted in a gradual decrease in PCDH17 expression, as evidenced by the division of one cell cluster into 9 distinct cell tracks based on their temporal sequence. Concurrently, the expression of the majority of immune-related gene set gradually diminishes during endothelial cell differentiation (**Figure 7**).

### Analysis of intercellular communication

**Figure 8:**
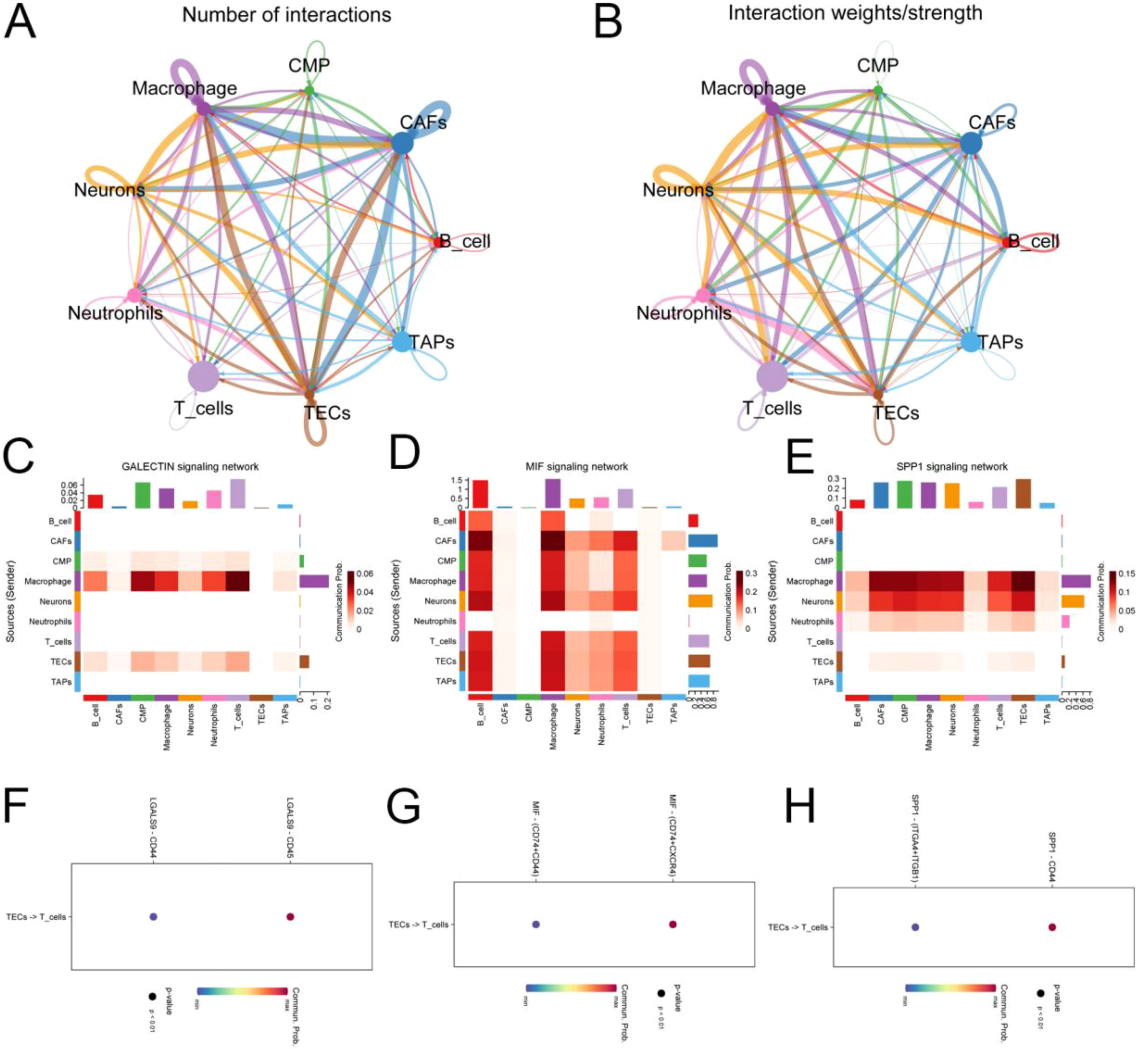
Analysis of cellular communication in the tumor microenvironment of pancreatic cancer. **(A, B)** Number and weight of intercellular interactions in the pancreatic cancer tumor microenvironment. **(C)** Heat map of intercellular GALECTIN signaling network interactions. **(D)** Heat map of intercellular MIF signaling network interactions. **(E)** Heat map of intercellular SPP1 signaling network interactions. **(F–H)** Target prediction of tumor-associated endothelial cells functioning on T cells. (TECs, tumor endothelial cells; TAPs, Tumor associated epithelial cells; CAFs, Tumor-associated fibroblasts).

The Cellchat R package was employed to assess the weights and probabilities linked to intercellular communication within the tumor microenvironment of pancreatic cancer. Application of the TCIA database for predicting immunotherapy outcomes in tumor patients. The differentiation of tumor-associated endothelial cells in pancreatic cancer exhibits polymorphic and multitemporal characteristics. We primarily aggregated genes from inflammatory signaling pathways in tumor-associated endothelial cells, with a focus on PCDH17, to delineate the differentiation trajectories within these cells. The primary interactions observed were between endothelial cells and T cells, mediated by signaling pathways such as GALECTIN, MIF, and SPPI (**Figure 8**).

### PCDH17 in endothelial cells involved in regulating the tumor microenvironment

**Figure 9:**
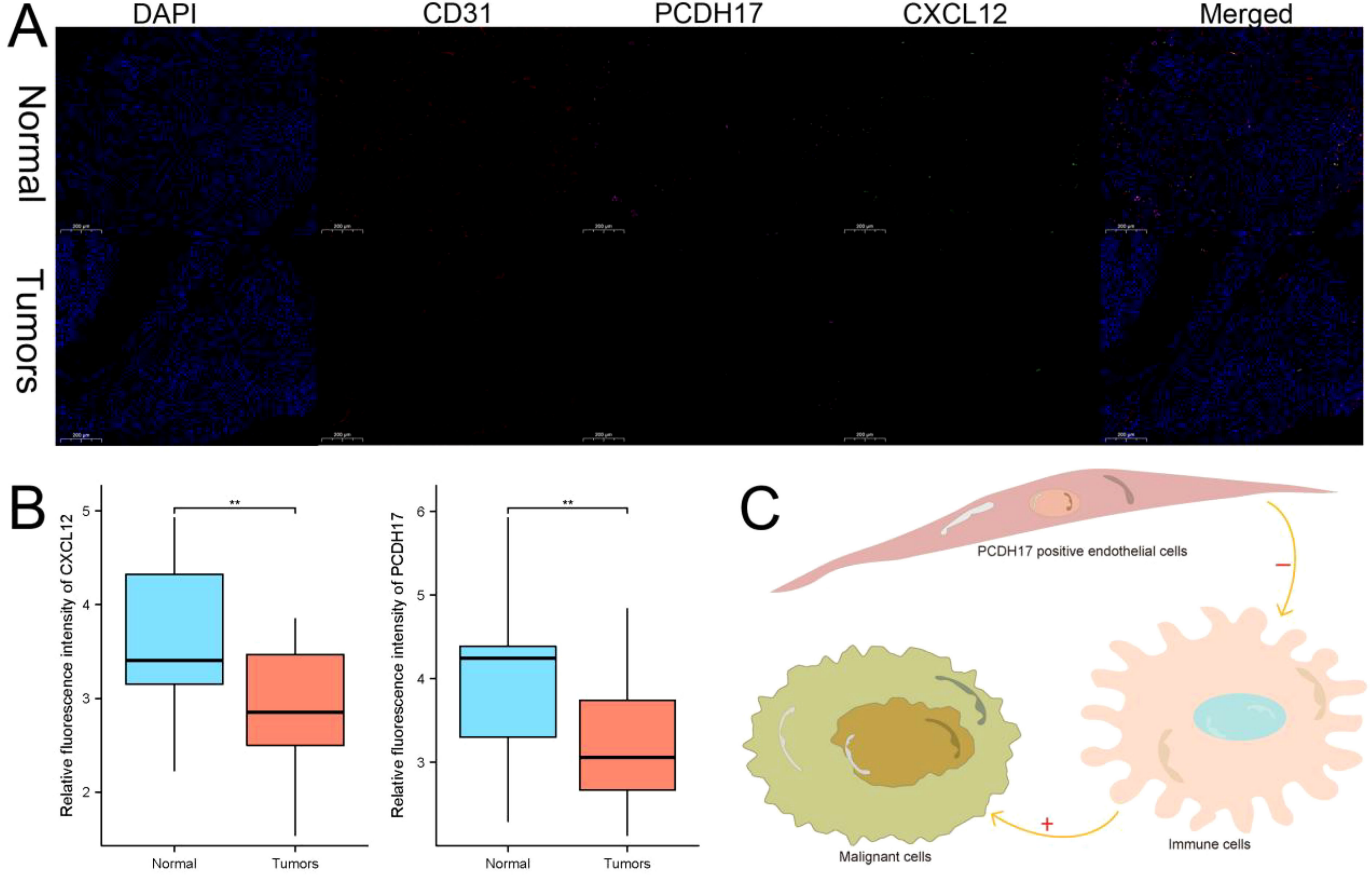
PCDH17 was expressed in endothelial cells and affected the tumor microenvironment. **(A)** PCDH17 was mainly expressed in endothelial cells. Both PCDH17 and CXCL12 expression was lower in tumor tissues than in paracancerous tissues. **(B)** Both PCDH17 and CXCL12 expression was reduced in pancreatic cancer tumor tissues. **(C)** PCDH17 involved in the regulation of the immune microenvironment function of pancreatic cancer tumors. ** denoting P < 0.01.

We employed immunofluorescence analysis to investigate the relationship between the expression localization and expression levels of PCDH17 and invasion-related genes. Our results clearly demonstrate that PCDH17 exhibits a significant colocalization pattern with the CD31 gene, indicating a potential regulatory interaction. PCDH17 demonstrates a notable level of co-localization with the gene CD31, which is commonly found in endothelial cells associated with tumors. Notably, there exists a strong correlation between PCDH17 and CXCL12 expression levels. The gradual decrease in CXCL12 expression aligns with the downregulation of PCDH17, consistent with our previous research findings (**Figure 9**).

## Discussion

The malignancy of pancreatic cancer is exceptionally high, rendering it the second leading cause of cancer-related mortality worldwide (26). The dismal prognosis of patients with pancreatic cancer primarily stems from the low rate of early diagnosis, susceptibility to postoperative recurrence, and high drug resistance (4). The adequacy of existing biomarkers for clinical diagnosis and treatment remains challenging. The tumor microenvironment plays a crucial role in determining the growth of malignant cells as well as the effectiveness of immunotherapy (27). The regulators of the tumor microenvironment have the potential to serve as therapeutic targets for pancreatic cancer.

Metabolic syndrome, including diabetes and obesity, is a prevalent risk factor for pancreatic cancer. The strong association between chronic subclinical inflammation triggered by metabolic syndrome and pancreatic cancer has been widely acknowledged (28). The findings revealed that metabolic syndrome not only hampers the secretion of anti-inflammatory factors but also stimulates the release of pro-inflammatory adipokines, primarily tumor interleukin (29). The continuous stimulation of chronic inflammation greatly increases the occurrence of pancreatic cancer in the body. At the same time, within the tumor microenvironment of pancreatic cancer, there is a complex interplay between inflammatory and immune responses to tumors (30). Firstly, the inflammatory response triggers the development of new cellular carcinomas (31). Additionally, the inflammatory response has the potential to modify the antigenic phenotype of tumor cells and actively contribute to their evasion from immune surveillance (32). The metabolic functions of stromal and immune cells are modulated through the influence of the inflammatory response, which in turn affects their intrinsic biological functionalities (33). In the immune evasion process of pancreatic cancer cells, inflammatory factors like IL-6, TNFα, and IL-10 have been found to be significantly involved. These factors play essential roles from the beginning of cancer cell formation to their progression and even during distant metastasis (34, 35).

This study reveals PCDH17 as a context-dependent regulator of tumor microenvironments across cancers. While upregulated in breast, cholangiocarcinoma, colon, and esophageal cancers, it is downregulated in cervical, renal, lung squamous, and pancreatic cancers, suggesting tissue-specific roles. Despite inconsistent expression trends, PCDH17 strongly correlates with 122 inflammatory factors and immune infiltration, particularly CD8+ T cells, implicating it in inflammatory TME modulation.

In pancreatic cancer, high PCDH17 expression associates with elevated inflammatory factors (TGF-β, IL6-JAK-STAT3, TNFA pathways) and enhanced immune activity, including antigen presentation and T-cell recruitment. Single-cell analysis confirms PCDH17’s predominant expression in endothelial cells, where its decline during differentiation parallels reduced immune gene activity. Cell-cell communication analysis highlights endothelial-T-cell interactions via GALECTIN/MIF/SPP1 pathways, with PCDH17 co-localizing with CD31 and correlating with CXCL12, a chemokine linked to immune recruitment.

Pancreatic cancer is characterized by a “cold tumor” phenotype, which significantly impedes the body’s specific immune response against tumor antigens (36). The process of cancer immune editing primarily occurs within the stromal cells residing in the tumor microenvironment, representing a pivotal mechanism employed by the immune system to effectively suppress both tumor growth and distant metastasis (16). Although cytokines and chemokines are primarily derived from inflammatory cells, biologically active small peptides produced by tumor cells can also elicit an inflammatory response. These mediators facilitate immune response crosstalk within the tumor microenvironment due to their close proximity, ultimately leading to a dampened cancer immune editing process (37). At the same time, the interaction between inflammation and immunity in the tumor microenvironment of pancreatic cancer leads to a suppressive environment that greatly hinders the infiltration of T-cells (38–40). Ultimately, the activation of regulatory T cells is induced in the context of pancreatic cancer due to the release of cytokines by mast cells within the tumor microenvironment, leading to immune tolerance (34, 41).

Procalcitonin, a transmembrane protein, plays an indispensable role as a mediator in the regulation of cell adhesion and downstream signaling pathways (42, 43). We present, for the first time, evidence of the involvement of PCDH17 in modulating the non-inflammatory tumor microenvironment in pancreatic cancer. Reduced expression of PCDH17 was observed in pancreatic cancer tissues. Intriguingly, the high PCDH17 group exhibited significantly elevated expression levels of inflammation-related gene sets compared to the low PCDH17 group. Based on these findings, we propose that PCDH17 regulates downstream inflammatory factors. The investigation of the tumor microenvironment in pancreatic cancer unveiled a noteworthy association between PCDH17 and T-cell infiltration. PCDH17 expression exhibited a decline within the tumor, resulting in a decrease in the abundance of T-cell infiltration. Additionally, there existed a robust positive correlation between PCDH17 and various genes responsible for immunosuppression. The diminished levels of PCDH17 within tumors significantly suppressed the expression of genes associated with immune checkpoint inhibition. Following analysis of single-cell data, it was observed that PCDH17 expression was primarily found in endothelial cells rather than immune cells. In comparison to normal endothelial cells, those within pancreatic cancer displayed a notable decrease in pathway activity related to the inflammatory response. Additionally, as endothelial cells underwent differentiation within the tumor microenvironment of pancreatic cancer, there was a further decline in PCDH17 expression. In the microenvironment of pancreatic cancer tumors, interactions between endoprosthetic cells and T cells occur through the MK signaling pathway, SPP1 signaling pathway, and GALECTIN signaling pathway. As a result, PCDH17 regulates the inflammatory response in the tumor microenvironment by inhibiting it through endothelial cells. Additionally, endothelial cells directly influence both T cell infiltration and suppression of immune function.

The present study entails a substantial workload; nevertheless, certain limitations remain. Firstly, the current investigation did not substantiate the inhibition of T cell differentiation through knockdown of PCDH17 in endothelial cells. Secondly, further exploration is warranted to elucidate the mechanism by which PCDH17 inhibits the inflammatory response. Lastly, validation of research findings in animal experiments is imperative.

## Conclusion

PCDH17 was predominantly expressed in endothelial cells and exerted an inhibitory effect on the inflammatory response, while also suppressing immune cell function. Consequently, PCDH17 exhibits potential as a target for regulating the immune-suppressive tumor microenvironment in pancreatic cancer.

## Data Availability

The original contributions presented in the study are included in the article/**Supplementary Material**. Further inquiries can be directed to the corresponding author.

## Supplementary material

Supplementary Table 1 Inflammatory gene set.

Supplementary Table 2 T-cell infiltration algorithm.
