# Supplementary material for: Single-cell and transcriptomic analyses reveal the role of PCDH17 in the non-inflammatory tumor microenvironment of pancreatic cancer
Source: Front Endocrinol (Lausanne). 2025 May 23;16:1559909. doi: 10.3389/fendo.2025.1559909 (PMC12141026; doi:10.3389/fendo.2025.1559909)
Supplement: Supplementary Table 1 — Inflammatory gene set. [file Table1.docx]

**Supplementary material**


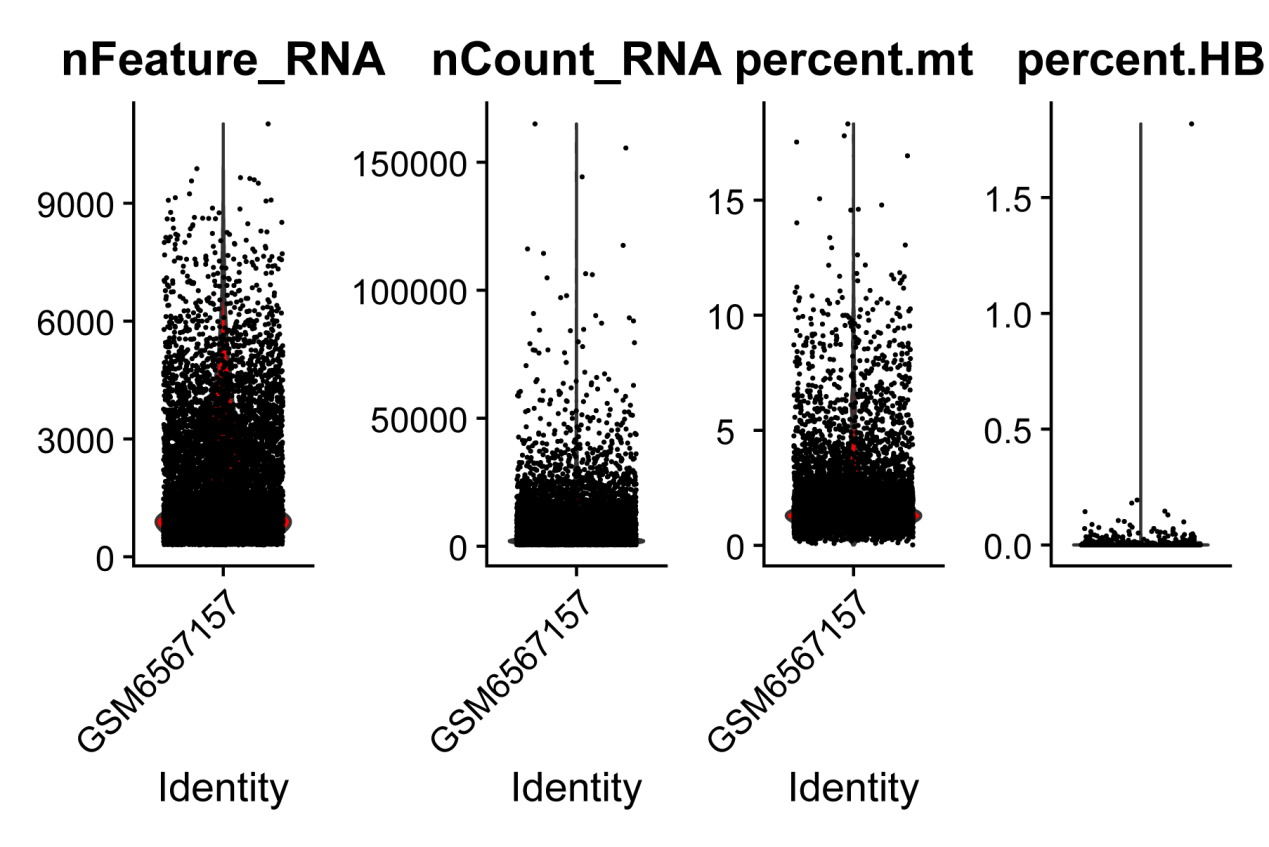


**Figure 1** Histogram of single-cell quality control.


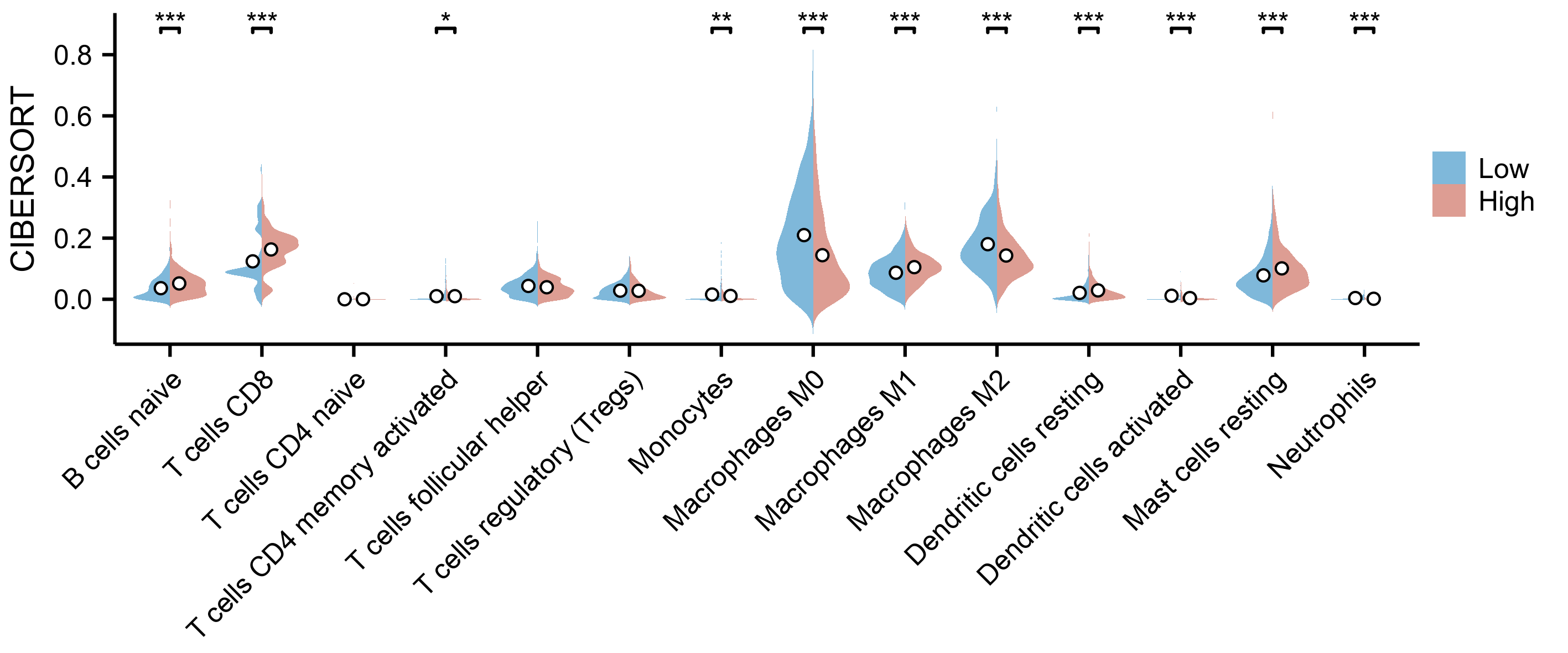


**Figure 2** Results of CIBERSORT algorithm analysis in pancreatic cancer patients.


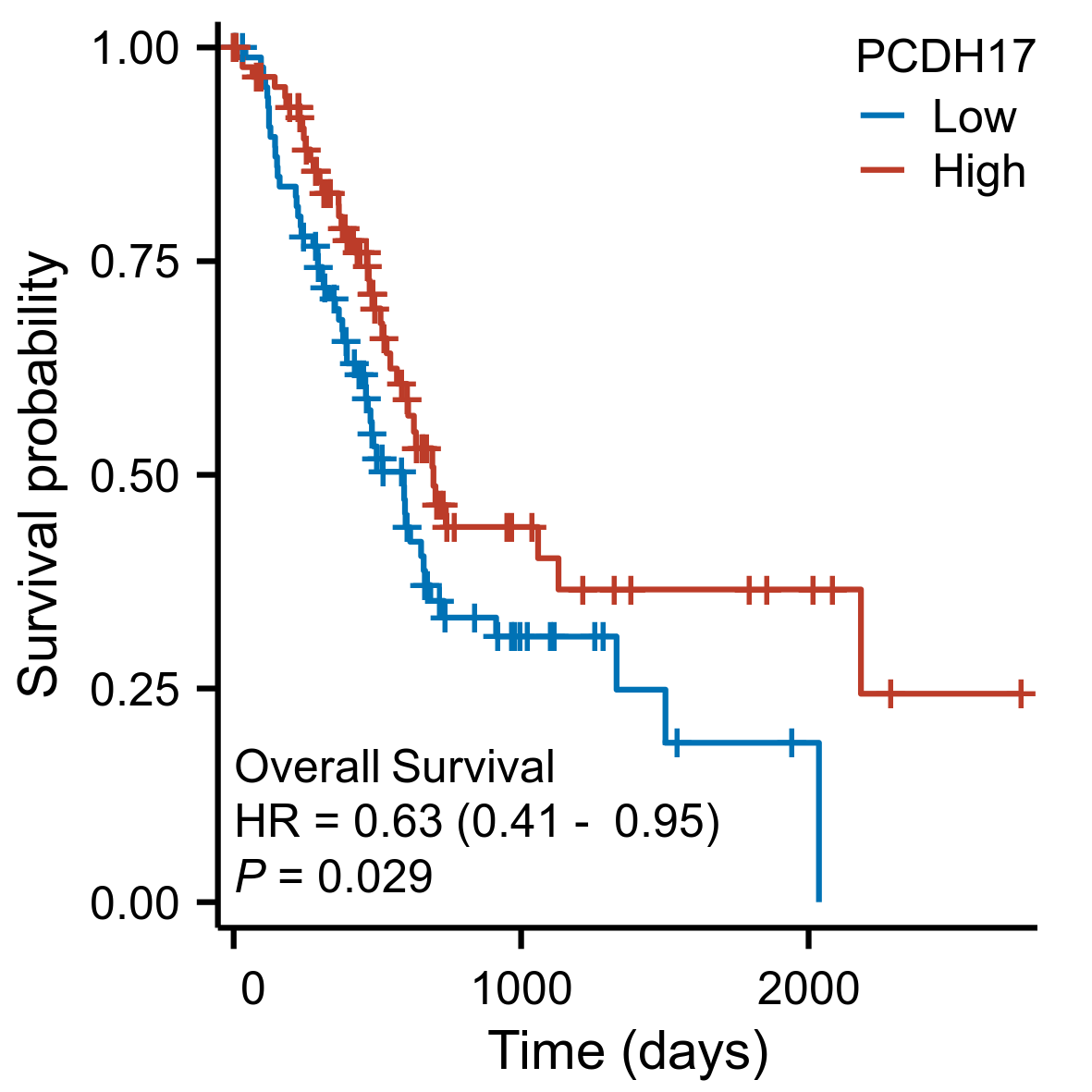


**Figure 3** Cox regression analysis was used to compare the prognostic value of PCDH17 in patients with pancreatic cancer.


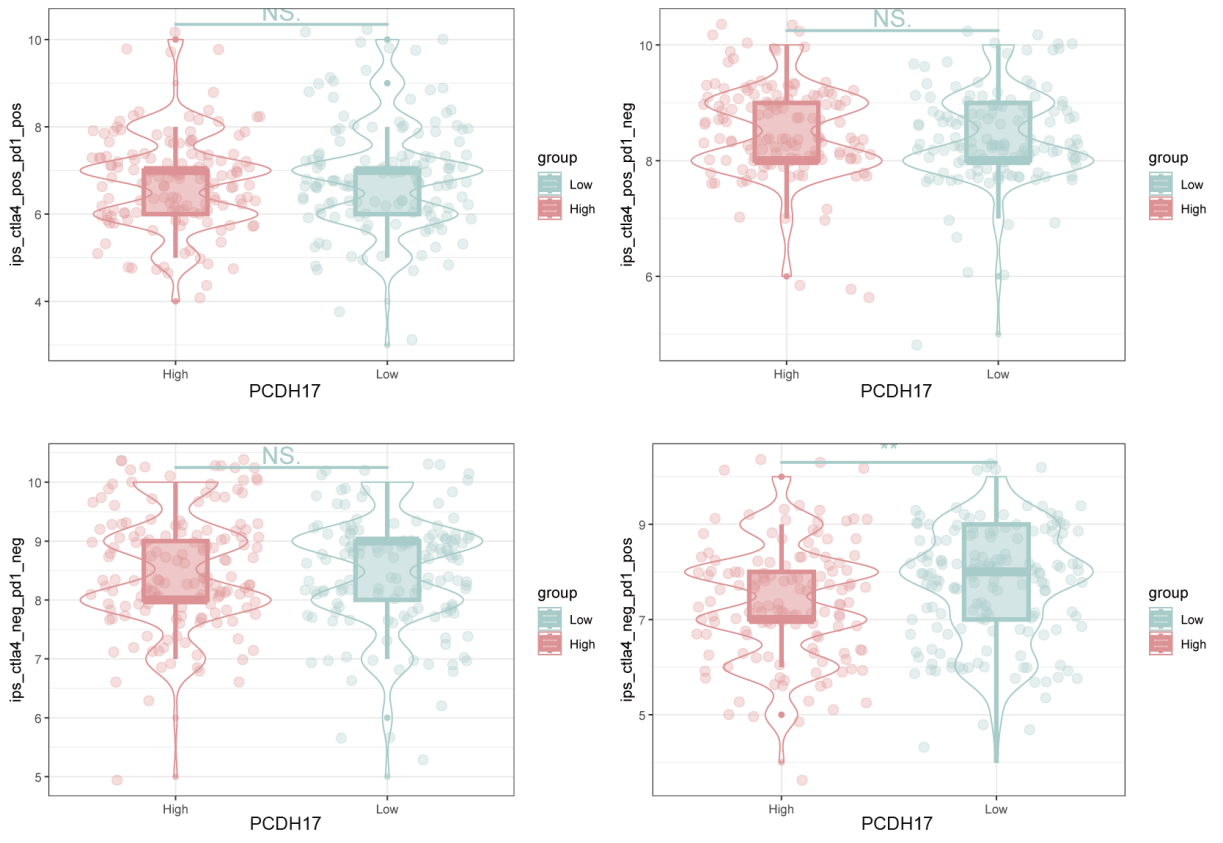


**Figure 4** Application of the TCIA database to predict immunotherapy outcomes in tumor patients.
