# Supplementary material for: Single-cell and transcriptomic analyses reveal the role of PCDH17 in the non-inflammatory tumor microenvironment of pancreatic cancer
Source: Front Endocrinol (Lausanne). 2025 May 23;16:1559909. doi: 10.3389/fendo.2025.1559909 (PMC12141026; doi:10.3389/fendo.2025.1559909)
Supplement: Supplementary file 3 [file DataSheet2.pdf]

Results of SCENIC analysis

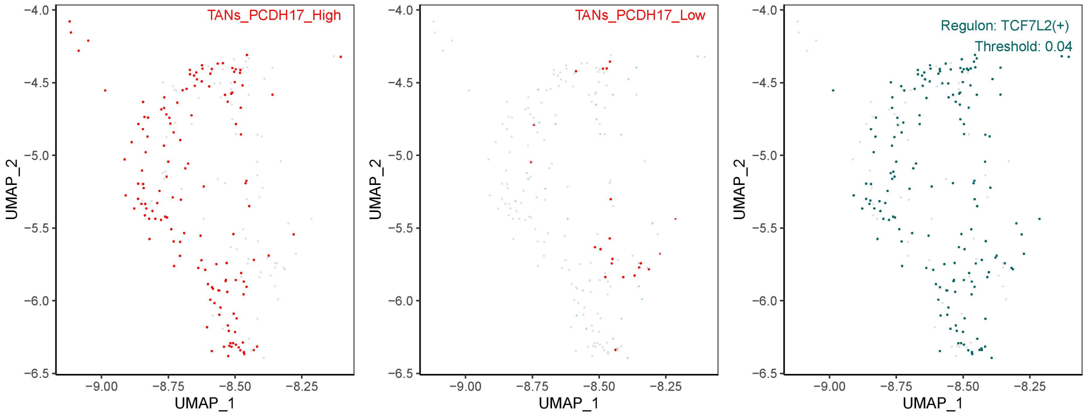

Figure 1 Analysis of TCF7L2 distribution in groups with high and low expression of PCDH17.

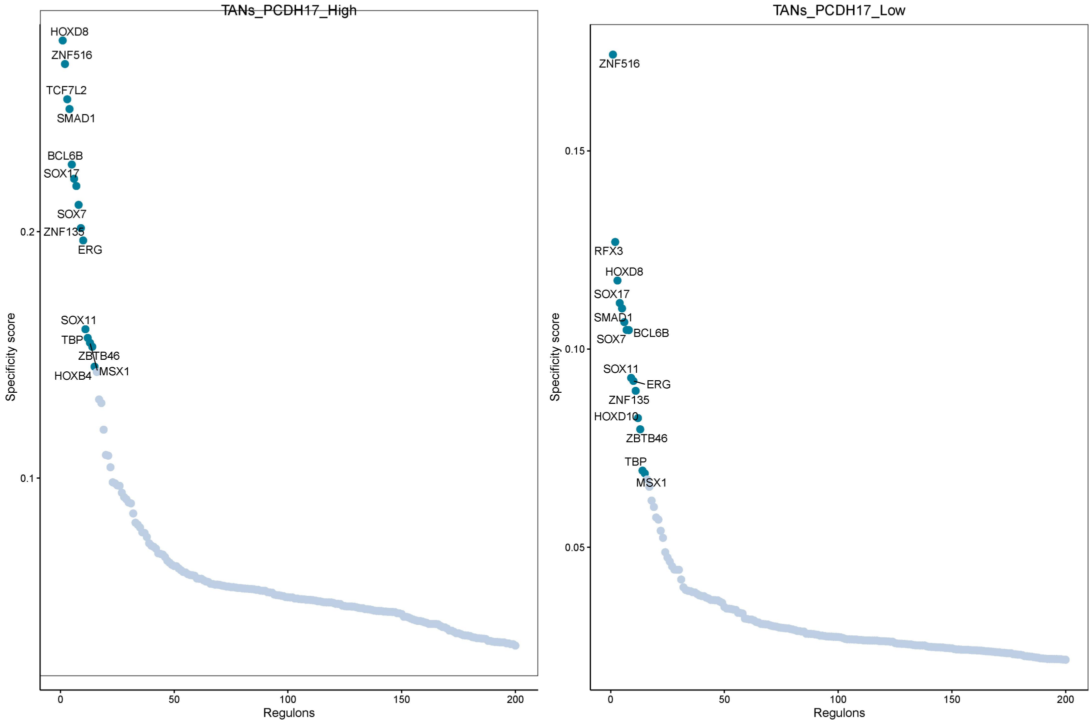

Figure 2 Analysis of the distribution of transcriptomic factors in PCDH17 high-expression and low-expression groups.

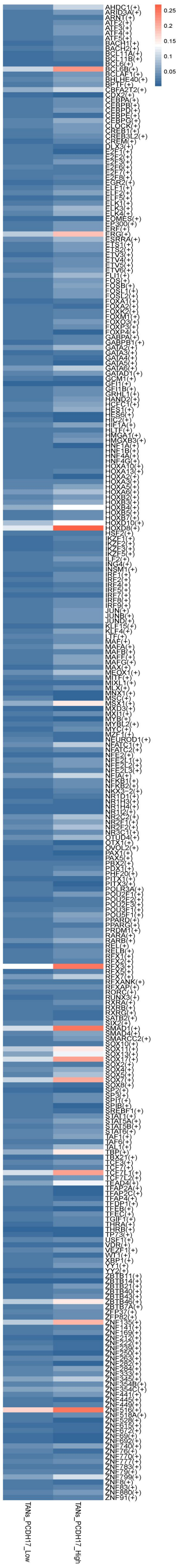

Figure 3 Heatmap illustrating the expression levels of transcription factors in the PCDH17 high-expression and low-expression groups.
